# Development of microsatellite markers for *Pinus maximinoi* derived from microsatellite-enriched libraries

**DOI:** 10.1186/1753-6561-5-S7-P2

**Published:** 2011-09-13

**Authors:** Ananda Virginia Aguiar, Camila C Mantello, Daiane Rigoni Kestring, Valderes Aparecida Sousa, Anete Pereira Souza, Laila Toniol Cardin

**Affiliations:** 1 Embrapa Forestry, Brazilian Agricultural Research Corporation, Colombo, Paraná, 83411-000, Brazil; 2 UNICAMP, Campinas, São Paulo, 13083-970, Brazil; 3 UNESP, Ilha Solteira, São Paulo, 15385-000, Brazil

## Background

Integrating the use of molecular techniques in ongoing breeding can help identifying divergent genotypes to be used in controlled crosses for the development of hybrids, estimating parentage and monitoring the efficient use of genetic variation. Breeding programs carried out at EMBRAPA have established a broad population genetic base for various species of pine, from which trees with high productivity and wood quality have been selected. *P. maximinoi* was included in this program as its timber has specific characteristics of great economic importance. One of the main features of *P. maximinoi* is its rapid growth, reaching 20 to 40 meters in height with a diameter at breast height of 100 cm. The species’ straight trunk with few branches is advantageous to the sawmill, and the timber’s thick fiber is necessary for quality paper production. IN this work we report on the initial steps of the development of microsatellite markers for *P. maximinoi* derived from microsatellite-enriched libraries. These markers will be used to characterize the breeding populations and germplasm collections of EMBRAPA.

## Methods

A microsatellite-enriched genomic library was constructed following the protocol described by [1]. The genomic DNA of *P. maximinoi* was digested with AFA*I* and enriched for two microsatellite motifs (CT)_8_ and (GT)_8_. Enriched fragments were amplified by PCR, ligated to a pGEM T-easy vector and transformed into competent XL1- blue *Escherichia coli* cells. The positive clones were selected using the B-galactosidase gene and then grown overnight in an HM/F medium with ampicillin. After PCR, 88 positive clones were sequenced in both directions. The sequences were assembled and edited in Seqman (DNAStar), and the repetitive regions were found using the Simple Sequence Repeat Identification Tool [2] Primer select (DNAStar) was used to design primer pairs flanking the microsatellite regions.

## Results and conclusion

Of the eighty sequences cloned only eight contained microsatellite sequences showing repeats and adequate flanking regions for primer design. The observed proportion of dinucleotide was 6.81%, while the tetranucleotide proportion was 2.27%. Seventy five percent of nucleotides were simple perfect repeats and 25 % were compost perfect. The explanation for this low yield (9.09 %) can be attributed to the genomic-enriching procedure. To overcome this problem the procedure will be repeated. The obtained sequences will be used for validation of *P. maximinoi* microsatellite primers. Eight microsatellite sequences of simply perfect and compost perfect nucleotides were observed for *P. maximinoi*. These markers will be validated and used to estimate the genetic diversity from the germoplasm collection of the Brazilian Agricultural Research Corporation (EMBRAPA).
